# Precision Medicine: Changing the way we think about healthcare

**DOI:** 10.6061/clinics/2017/e723

**Published:** 2018-11-23

**Authors:** Gustavo Rosa Gameiro, Viktor Sinkunas, Gabriel Romero Liguori, José Otavio Costa Auler-Júnior

**Affiliations:** I Faculdade de Medicina FMUSP, Universidade de Sao Paulo, Sao Paulo, SP, BR; II Laboratorio de Cirurgia Cardiovascular e Fisiopatologia da Circulacao (LIM-11), Instituto do Coração (InCor), Hospital das Clinicas HCFMUSP, Faculdade de Medicina, Universidade de Sao Paulo, Sao Paulo, SP, BR; III Divisao de Anestesiologia, Hospital das Clinicas HCFMUSP, Faculdade de Medicina, Universidade de Sao Paulo, Sao Paulo, SP, BR

**Keywords:** Precision Medicine, Medical Education, Molecular Biology, Genetics, Genomics

## Abstract

Health care has changed since the decline in mortality caused by infectious diseases as well as chronic and non-contagious diseases, with a direct impact on the cost of public health and individual health care. We must now transition from traditional reactive medicine based on symptoms, diagnosis and treatment to a system that targets the disease before it occurs and, if it cannot be avoided, treats the disease in a personalized manner. Precision Medicine is that new way of thinking about medicine. In this paper, we performed a thorough review of the literature to present an updated review on the subject, discussing the impact of the use of genetics and genomics in the care process as well as medical education, clinical research and ethical issues. The Precision Medicine model is expanded upon in this article to include its principles of prediction, prevention, personalization and participation. Finally, we discuss Precision Medicine in various specialty fields and how it has been implemented in developing countries and its effects on public health and medical education.

## INTRODUCTION

To live longer, yet remain young and healthy, is one of the most ambitious dreams of humanity 1. At the beginning of the 20^th^ century, according to the World Health Organization, the global average life expectancy was approximately 35 years. By the end of the century, the average human lifespan had almost doubled 2. This was mainly due to advances in science and technology leading to improved sanitary conditions, new therapies and drugs (such as antibiotics), new imaging methods (starting with the X-ray, which won the Nobel Prize in 1901), and the introduction of preventive medicine including vaccination campaigns and, later in the century, the promotion of healthy lifestyles 3,4.

**Figure 1:**
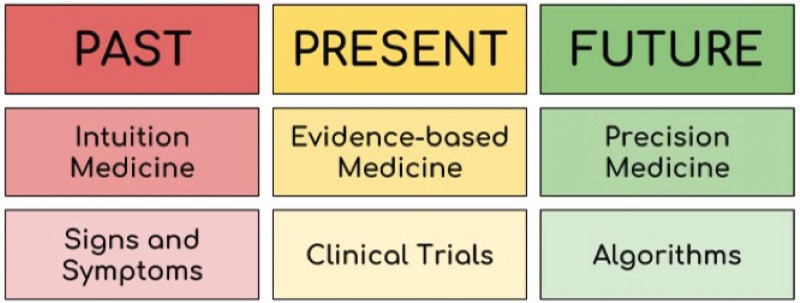
The evolution of medicine. In the past, medicine was practiced according to the signs and symptoms presented by the patient and was solely based on the individual knowledge of the physician and thus was called intuition medicine. Currently, medicine is based on the evidence produced by scientific research, including clinical trials, which is designated as evidence-based medicine. In the future, medicine will be practiced according to algorithms that will take into consideration the patient's characteristics, such as their genome, epigenetics, and lifestyle, constituting precision medicine.

Standing at the peak of healthcare evolution, one might wonder if this increase in life expectancy will continue. Moreover, within the current limits of the lifespan, we are already paying the price for living longer. Currently, we spend approximately 50% of the budget dedicated to healthcare on treating terminal illnesses such as cancer, heart failure, and other degenerative diseases 5,6. The single factor common to all of these diseases is aging. To better target the issues related to this increase in life expectancy, medicine in the 21^st^ century must focus on attaining the 4 Ps stated by Dr. Leroy E. Hood: prediction, prevention, personalization, and participation 7–9. We must now transition from traditional reactive medicine based on symptoms, diagnosis and treatment to a system that targets the disease before it occurs and, if it cannot be avoided, treats the disease in a personalized manner. This evolution in medicine is summarized in Figure 1.

## METHODS AND RESULTS

**Figure 2:**
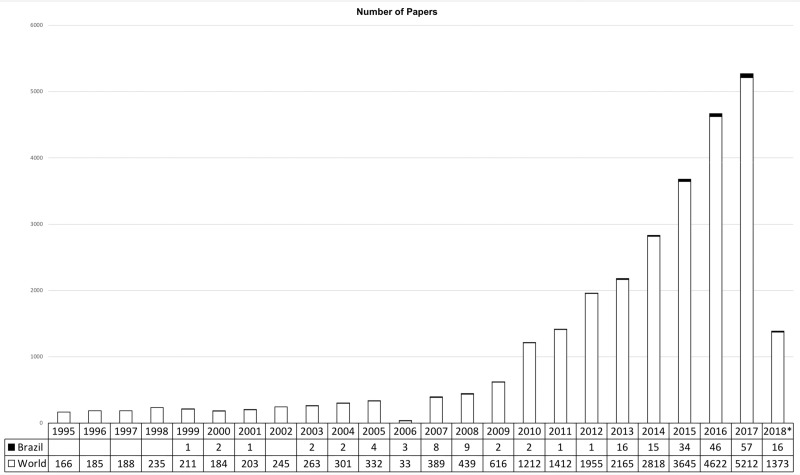
Graph showing the number of papers on “precision medicine” in the PubMed/MEDLINE database worldwide and the Brazilian contribution. *Articles until March 2018.

Illustrating the increasing popularity of “precision medicine”, a literature search of the PubMed/MEDLINE database conducted in March 2018 revealed 29,884 articles worldwide on the topic but only 241 when we added the “Brazil” filter. The number of articles has increased exponentially during the past decade (Figure 2), with half of these articles published in the last three years.

In addition, a meticulous search was performed for “precision medicine” in the PubMed/MEDLINE database from the same time period. Articles that referred to a specific disease or medical technique were excluded, as the authors agreed to focus on the precision medicine concept. Similar articles cited by the ones found in the initial search were also included. Those papers formed the database used to write a narrative literature review on the field.

## DISCUSSION

### The health disease process: from classical prevention theories to the 4 Ps

Reactive medicine is based on the principle of only treating diseases after their clinical initiation. This way of thinking has been found to be economically inferior to a model that acts before the beginning of symptoms in an effort to disrupt symptom development, which is known as Preventive Medicine 10.

Preventive medicine was clearly an important step for reducing the burdens of various diseases in populations. Preventive interventions focus on risk factors associated with a pathology - for instance, tabagism and the incidence of lung cancer – to generate a number needed to treat in order to prevent a disease event 10. In that sense, preventive medicine focuses on populations, whereas precision medicine includes tailored treatments and preventions.

We are rapidly approaching a point in healthcare evolution where it is possible to treat a disease while considering the various factors that affect individual patients so that they may receive customized care. We are transitioning from an age of absolute gold standards to one in which generalizations give way to patient-specific diagnostics and therapeutics. As it relates to predictive medicine, technology is allowing us to better understand not only our genomic DNA but also our epigenetic response to environmental changes, the resulting proteome in each of our cells, and the post-translational modifications affecting our proteins. We are delving deeper into the analysis of proteomics, transcriptomics, genomics, metabolomics, and lipidomics, which will allow us to precisely predict and target diseases 11.

The concept of participatory medicine rests on the idea that patients should play a decisive role in their own healthcare by actively controlling their health status and participating in the decision-making process regarding their treatments. Doctors are now invited to evaluate treatment possibilities while considering the benefits and limitations of each alternative, rather than simply imposing a treatment as before. With more information available, decision-making will become a much more complicated process, given the increasing number of variables that must be taken into consideration.

Finally, the fourth P, which stands for “personalized medicine” has since 2011 given rise to the term “precision medicine”. This resulted from a publication of the National Research Council arguing that “precision” is a better term for referring to the classification of people into subpopulations using their common genetic patterns, lifestyles, drug responses, and environmental and cultural factors. Thus, precision medicine is an innovative approach in which enormous amounts of information produced every day in the healthcare system are used to deliver the most efficient treatment or preventive care, at the right time, to the patients who will best benefit from it. The proposal is not to design a unique therapeutic intervention for each patient, such as a personalized one-patient drug, which would be financially and technically difficult. Precision medicine could mean higher medication prices for specific subgroups of patients, but it is important to understand that it is a way to avoid the costs of unnecessary and inappropriate treatments for individuals not responsive to specific therapeutic approaches.

The possibility of characterizing individuals using their genetic polymorphisms has become available due to the development of inexpensive and rapid DNA sequencing methods over the last decade 11. Ten years ago, sequencing the genome cost US$ 1 billion and took 13 years; today it costs US$ 1,500 and takes only a few hours. At the same time, we have seen improvements in data storage technologies that allow us to register an enormous amount of data, e.g., in 2017, the run rate for global IP traffic is estimated as 1.4 zettabytes (1 zettabyte=10^2^^1^ bytes) per year 12. The discovery and collection of various types of information - from cellular signaling pathways to clinical trials data - can now be used to facilitate the identification and targeting of several diseases. These developments produced a comprehension of disease pathophysiology that is incompatible with the current and limited International Classification of Diseases (ICD). Taking into account the need to reorganize diseases by considering their biomolecular aspects, the National Research Council suggested the creation of a new taxonomy system, namely, the Knowledge Network of Disease 13. This new taxonomy should integrate clinical information and research data as layers of information including data regarding the genome, microbiome, epigenome, exposome, and signs and symptoms of specific populations.

### Implication of the 4 Ps theory in clinical research and its promotion in developing countries

Application of the precision medicine theory in developing countries is sometimes considered uncertain 14. In addition to the problem of financial support for the development of new therapies, the population of these countries remains without a sufficient amount of collected genomic data. For example, India has 20% of the global population but represents only 1% of existing genetic data 15. This representation problem also exists in genetic research. In genome-wide association studies (GWAS), 96% of the subjects studied were of European descent, which can result in findings that are less relevant for populations in developing countries 16.

Another challenge in developing countries is the gap between global health research funding and the health problems that affect populations from undeveloped and developing countries. According to the Global Forum for Health Research of 1999, only 10% of global funding goes to research on diseases that affect 90% of the world's population 17. Furthermore, the number of publications in low- and middle-income countries is low; from 1990 until 2000, publications from all the countries of Latin America represented only 5.45% of the publications of the United States 18.

### Precision medicine and medical education

Innovative ways to practice medicine will lead to redesigning the way in which we teach medicine. Medical education will need to shift the focus from pure content to the development of integrative skills and competences.

Gordon Moore, co-founder of Intel, postulated in 1965 that the number of transistors in a dense integrated circuit, and thus the processing capacity of computers, would double every 18 months. Scientific knowledge, particularly in the medical field, seems to be following a similar trend, primarily due to the computer revolution itself. This, although desirable, brings with it huge challenges to medical education, since no one person can follow all of the rapid and critical changes occurring in medicine every day. Therefore, medical students may often find themselves lost in a series of articles, textbooks, news stories, and editorials. However, it is crucial that they develop the ability to critically select information that is relevant for their education and apply this knowledge to solve the everyday challenges of various situations. However, basic subjects such as molecular and cell biology, genetics, and pathophysiology are more important than ever to understanding and practicing precision medicine.

Another competence of great importance to the medical education of the next era is interpersonal skills. Doctors are required, now more than ever, to be able to communicate and discuss treatment options not only with their patients but also with a series of other professionals with whom they must cooperate, both internally and externally in the healthcare sector. These other professionals include nurses, physiotherapists, speech therapists, and occupational therapists, as well as engineers and physicists, among others. It will also be beneficial for the next generation of doctors to understand, at least at a basic level, subjects that are not included in the current medical curriculum, such as coding, artificial intelligence, blockchain technology, 3D printing and a series of other related topics.

Regarding how precision medicine could be taught to medical students, we believe that two main adjustments should be made to the current medical curriculum: first, learning of basic science subjects, focusing on how this knowledge can directly impact clinical practice, should be emphasized; second, it is of great importance to promote discussion panels on the evolution of medicine, bringing in experts in the groundbreaking fields that are changing healthcare and, thus, stimulating students to pursue a career on these areas. The University of Sao Paulo Medical School is developing new curricula that integrate basic and clinical sciences to include the skills required for the future of medicine.

### Applications of precision medicine

#### What is already happening: The cases of oncology and psychiatry

Cancer is a leading cause of death worldwide, accounting for 8.8 million deaths in 2015 19. Research examining the mechanisms involved in the pathogenesis of cancer, including the understanding of oncogenes and epigenetic clues in oncogenesis, has been performed continuously and at an increasing pace over the last several decades 20. Therefore, the discovery of potential targets for drug development - aiming to block the expression of those oncogenes by inactivating them or their pathways - is one of the promising aspects of precision medicine. One example of this concept, which is already being clinically applied, is the treatment of chronic myeloid leukemia (CML). Allogeneic bone-marrow transplants had long been considered the best treatment option, but they were indicated only for younger patients and continued to cause significant mortality 21. However, the discoveries of molecular predispositions that generate a variety of diseases, such as CML, made possible the development of more specific medications aimed at new molecular targets. Currently, the administration of a competitive inhibitor of the Bcr/Abl tyrosine kinase - an oncoprotein expressed in 95% of patients with CML and the molecular target for this disease - is able to achieve an 80% success rate of a complete cytogenetic response in newly diagnosed patients 22.

Among psychiatric illnesses, post-traumatic stress disorder (PTSD) is being extensively studied in the USA due its prevalence in soldiers returning from combat, which can reach 18.5% in this population according to data of Vietnam veterans 23. Animal research has already shown that in a stressful environment, epigenetic alterations in the hypothalamic-pituitary-adrenal axis can modulate the stress response 24. Other research has shown that combat veterans present an epigenetic alteration (hypomethylation) in the exon promoter region *NR3C1*-F1 of the glucocorticoid receptor gene, which could explain the changes in the neuroendocrine response of these soldiers 25. In contrast, the same exon *NR3C1*-F1 is the site of hypermethylation in suicide victims with a history of childhood abuse 26. These two subpopulations from different stress environments may be seen comparable if only the signs and symptoms are considered. The knowledge that these groups present opposing epigenetic modifications to the same gene makes it possible to approach treatment of these patients while taking into consideration their particular disease mechanisms and developing more efficient treatments for each group.

#### What is going to happen: Tissue engineering and regenerative medicine

Tissue engineering and regenerative medicine are fields that can make important contributions to developments in precision medicine. Among the innumerable possibilities within these research areas, there are two topics of interest: organs-on-a-chip and personalized stem cell therapies.

Organs-on-a-chip are 3D tissue-engineered platforms with microfluidic network systems that can be used to study the effect of virtually any substance on a specific tissue 27,28. This technology has gained great interest mainly due to the limitations inherent to the use of animal models during safety and efficacy testing, such as the poor translational potential and the ethical issues raised by animal experimentation 29. Although organs-on-a-chip have been increasingly utilized for preclinical testing of drugs, they present an even more interesting potential for use in precision medicine. The creation of patient-specific organs-on-a-chip would allow testing drug therapies *in vitro* before proceeding to the actual clinical treatment 30. Using induced pluripotent stem cells (iPSCs) from adult tissue, such as skin or blood, and differentiating them into any target tissue, from liver cells to neurons, it would be possible to create a patient-specific organ-on-a-chip to study how different drugs act on the patient's own tissue and, based on this screening, to define a better therapeutic approach to treat a given disease, which can also be simulated *in vitro* 31–33. The New York Stem Cell Foundation (NYSCF) recently took an important step toward making personalized organs-on-a-chip closer to clinical application by creating a high-throughput robotic platform to automate the process of transforming patient samples into iPSCs and later into the target tissue cell types 34. The final development of the organs-on-a-chip technology would be the creation of a whole body-on-a-chip, thus allowing the investigation of not only a drug's efficacy on the targeted tissue but also its adverse effects on other tissues of the patient 35.

Personalized stem cell therapies, in turn, join the potential of regenerative medicine, which aims to replace or regenerate cells, tissues and organs, to the tailored approach used in precision medicine. While stem cell therapies are already, by default, a type of personalized therapeutic approach, since cell sources are, in most of the cases, autologous, methods exist to make these treatments even more personalized. The most interesting is the use of gene editing technologies to enhance stem cells or cure genetic disorders before the delivery of cells into the patient. Briefly, the process is characterized by isolating patient cells from any tissue, reprogramming them into iPSCs, modifying or correcting the cell genotype (or epigenetic factors) using technologies such as Zinc finger nucleases (ZFN), Transcription activator-like effector nucleases (TALEN) and Clustered Regularly Interspaced Short Palindromic Repeats/CRISPR associated protein 9 (CRISPR/Cas9), differentiating them into the desired tissue and implanting them back into the patient 36,37. Some of the best examples of the potential of this approach are in the treatment of hematological diseases 38,39. While clinical trials are still not a reality, the technique has already been demonstrated to be successful for the treatment of sickle cell disease 40 as well as the correction of β-globin gene mutations in subjects with thalassemia 41–44. Advances in gene editing technologies, together with the improvements in iPSCs generation, will soon allow a wide range of diseases to be targeted by personalized regenerative medicine approaches.

#### Initiatives in precision medicine worldwide

The development of algorithms to predict and treat diseases based on a subpopulation-specific set of characteristics, such as genetics, drug responses, lifestyles, and social demands, requires a vast amount of information. Thus, instead of performing a series of separate and independent research protocols, initiatives aiming to create and integrate data from different medical centers are necessary. Using currently available tools, such as cloud computing, artificial intelligence, and big data, the collection and analysis of different databases will allow the creation of algorithms to direct clinical practice.

To contribute towards the development of precision medicine, the National Institutes of Health (NIH) announced, in 2015, the Precision Medicine Initiative (PMI), a program for delivering resources to projects aimed at creating new methods to improve healthcare by applying technologies that maximize effectiveness by taking into account individual variability in genes, environments, and lifestyles. The initiative focused not only on cutting-edge technologies but also on the interdisciplinary context of the research topics. Since then, researchers of various fields have been working together to break through this new frontier of medicine 45–47. The California Initiative to Advance Precision Medicine (CIAPM) is another initiative, launched in 2015, aiming to build a centralized information base. This initiative, a partnership among the University of California, the state of California and other entities, is expected to stimulate collaborations among clinicians, scientists, and patients to improve healthcare outcomes and foster innovations in the biomedical field.

One of the issues involved in building these large and centralized databases is related to consent, confidentiality and intellectual property. An immense ethical challenge exists in organizing these databases and giving access to different clinicians and researchers while protecting patient information from third party interests, as well as protecting the intellectual property of the scientist or clinician who collects the data.

Although the problem of limited genomic data from populations of developing countries is mainly due to the high costs, several startup companies are seeking to increase the amount of genomic data generated from Asian populations. The GenomeAsia 100K, a nonprofit consortium of companies and academics, wants to create reference genomes of all major Asian ethnic groups starting with the sequences of 100,000 people.

Finally, it is important to note that the significant advances in precision medicine produce concerns that must also be considered. Again, ethical issues are a critical problem, considering that all of the genetic, environmental, and lifestyle information of each patient will be stored in a database in the future. How should this information be kept safe and confidential? Taking this concept one step further, the possibility to genetically engineer patients and therefore create “super-humans” is not far away and fuels heated ethical debates. Considering this possibility, diseases will be able to be predicted, and we will also have the tools to prevent them before they occur; addressing this possibility is not something we are currently used to and is thus another skill we still need to develop.

## AUTHOR CONTRIBUTIONS

GRG and VS contributed to the conception and design of the article, literature review, drafting the article, critical revision of the article and final approval of the version to be published. GRL contributed to the literature review, drafting the article, critical revision of the article and final approval of the version to be published. JOCA Jr. contributed to the conception and design of the article, critical revision of the article and final approval of the version to be published.
